# Successful Oocyte Retrieval, Fertilization, and Clinical Pregnancy with Low Serum *β*-hCG on the Day of Oocyte Collection: A Reappraisal of the Definition of the Empty Follicle Syndrome

**DOI:** 10.1155/2020/9210651

**Published:** 2020-02-08

**Authors:** Nicole A. Van De Velde, Charalampos Chatzicharalampous, Awoniyi O. Awonuga

**Affiliations:** ^1^Resident in Obstetrics and Gynecology, University of Tennessee Health Science Center, USA; ^2^Division of Reproductive Endocrinology and Infertility, Department of Obstetrics and Gynecology, NYU School of Medicine, NYU Langone Reproductive Specialists of New York, USA; ^3^Division of Reproductive Endocrinology and Infertility, Wayne State University School of Medicine, USA

## Abstract

**Objective:**

To describe a case of successful oocyte retrieval, fertilization and clinical pregnancy despite very low *β*-hCG level, twelve hours after ovulation trigger.

**Design:**

Case report. *Setting*. Academic medical center. *Patient*. A 38-year-old patient inadvertently administered 2,000 IU hCG for final oocyte maturation; serum hCG twelve hours later was 16 IU/L. *Interventions*. Effort to obtain and administer a booster dose of hCG over the next twenty-seven hours failed. *Main Outcome*. Successful oocyte retrieval.

**Results:**

Fourteen oocytes were retrieved of which twelve were in metaphase II and nine fertilized after intracytoplasmic sperm injection (ICSI). Of these, eight embryos survived to day 5 and were subjected to preimplantation genetic screening (PGS) by comparative genomic hybridization (CGH). Results were available the next day, three of the embryos were euploid and one was transferred on day 6. Pregnancy was confirmed twelve days later and currently the patient has an ongoing singleton intrauterine pregnancy.

**Conclusion:**

Reproductive Endocrinology and Infertility specialists should be aware that final oocyte maturation could occur following injection of a lower dose of hCG with excellent fertilization rate and embryo development.

## 1. Introduction

Assisted reproductive technology is now an established and successful treatment for infertile couples. Assisted conception units have incorporated teaching sessions to educate patients as to what to expect and what their responsibilities are during treatment. Despite this, patients inadvertently get things wrong.

One such situation occurs when patients fail to administer the correct dose of hCG or inject saline only instead, for final oocyte maturation prior to ultrasound directed follicle aspiration. This may lead to failure to retrieve oocytes from mature follicles in the face of appropriate estradiol (E2) levels despite repeated aspiration and flushing [1] a phenomenon termed empty follicle syndrome (EFS).

The pathophysiology of inability to retrieve oocytes is due to an inadequate level of serum *β*-hCG. An adequate level of serum *β*-hCG is required to promote the detachment of the cumulus-oocyte complex (COC) from the follicular wall allowing COC to freely float in the follicular fluid, from where it can be aspirated [2, 3]. This phenomenon is called “false empty follicle syndrome (FEFS)” as against its other counterpart called “genuine empty follicle syndrome (GEFS)” defined as adequate serum *β*-hCG (>40 IU/L) and a lack of oocytes retrieved from mature follicles [4]. “False empty follicle syndrome” therefore is the inability to retrieve oocytes when *β*-hCG level is <40 IU/L immediately before or during oocyte retrieval.

However, different authors have reported a wide range of serum *β*-hCG levels (5 to 161 IU/L) on the day of oocyte collection as the minimum *β*-hCG concentrations consistent with adequate hCG triggering [4–8]. One group [9] went further, stating that serum *β*-hCG of <10 mIU/mL is 100% sensitive, specific, and predictive of EFS. Live births have resulted after administration of a rescue course of hCG and repeat oocyte retrieval 24-36 h later in the setting of false [9–11] and genuine [12] empty follicle syndrome.

Here, we report the case of a 38-year-old patient who inadvertently administered 2,000 IU of hCG for final oocyte maturation and her serum *β*-hCG 12 hours later was 16 IU/L. Effort to obtain and administer a booster dose of hCG over the next 27 hours failed. Thirty-nine hours after the initial administration of the hCG trigger, a decision was made to proceed to egg collection with the understanding that if oocytes were not retrieved after aspiration of a reasonable number of follicles from one ovary the procedure would be abandoned until a booster dose of hCG could be administered, and oocyte collection was postponed accordingly. Oocytes were retrieved and fertilized by ICSI with the transfer of one euploid blastocyst on day 6, resulting in an ongoing singleton pregnancy.

## 2. Materials and Methods

### 2.1. Case Report

The patient is a 38-year-old female who provided informed consent to report clinical details and data of the case. Her past medical history includes polycystic ovarian syndrome (PCOS), endometriosis, and tubal and male (partner with testicular cancer and right orchidectomy) factor infertility. She underwent controlled ovarian hyperstimulation for in vitro fertilization (IVF) using a GnRH antagonist protocol. Superovulation was with follitropin alfa (Gonal F) and menotropins (Menopur) for 10 days. Two hundred and fifty micrograms of Ganirelix acetate daily was added on day six to prevent a premature LH surge. On day 10, there were 10 follicles that measured 16 mm or greater, and the serum estradiol level was 2,818 pg/mL. The decision was made to proceed with hCG trigger, and the patient was instructed to inject a dose of 10,000 IU intramuscularly.

The patient had attended our mandatory IVF class and verbalized understanding of the instructions, which included aspirating 1 mL of saline solution from the 5 mL vial and injecting it into the vial of sterile dried powder of hCG (10,000 usp units per vial).

The patient, however, inadvertently aspirated the entire 5 mL of saline and injected the entire volume into the 10,000 IU vial of hCG, mixed the solution, aspirated 1 mL of the solution, and administered this to herself. Thus, the patient only injected 2,000 IU of hCG instead of the prescribed 10,000 IU. The patient did not volunteer this information until she was confronted, which occurred after her serum *β*-hCG level came back at 16 IU/L twelve hours after she administered her hCG trigger.

It is our practice to routinely draw blood for serum *β*-hCG 12 hours post-hCG administration to detect errors in hCG administration as in the case presented. Unfortunately, this occurred on a Sunday and efforts made to obtain and supply the patient with a supplemental rescue dose of hCG proved abortive. This effort continued until Monday morning without success.

After much discussion, we decided to proceed with the egg collection as 39 hours had passed since her initial hCG trigger. The plan was to attempt to empty a reasonable number of follicles in one ovary, and if no oocytes were retrieved to stop the procedure, give a booster hCG later that Monday morning, and have the patient return the following day to complete the oocyte retrieval procedure. At the attempted retrieval, oocytes were retrieved after emptying the first few follicles; hence, the retrieval was completed.

## 3. Results

We retrieved fourteen oocytes out of eighteen follicles, twelve of which were in metaphase II and nine fertilized after intracytoplasmic sperm injection (ICSI). Eight embryos survived to day five. The patient and her partner had requested preimplantation genetic testing of their embryos. Of the eight embryos biopsied and tested, three were euploid and one was transferred on day six. The patient was confirmed pregnant twelve days later and now has an ongoing singleton intrauterine pregnancy at the time of this report.

## 4. Discussion

False empty follicle syndrome is a rare event the etiology of which is attributed to errors in hCG administration at the time of ovulation trigger. We report a case in which clinical pregnancy resulted when the serum *β*-hCG level twenty-four hours before oocyte retrieval was 16 IU/L (12 hours after the patient inadvertently administered the wrong hCG dose) in which effort to administer an additional hCG trigger dose failed. Typically, a threshold of 40 IU/L is used 36 hours after hCG administration to ensure oocytes are collected at time of retrieval. Others have suggested a serum *β*-hCG level of 20 IU/L on the day of retrieval as the threshold to verify that the correct dose of hCG has been administered, since this is the serum level that will result in a positive urinary pregnancy test [13]. The level of serum *β*-hCG on the day of egg collection below which an attempted oocyte retrieval will result in an empty follicle is reported as <12 IU/L [9, 14]. However, Ndukwe and colleagues [9] went further stating that a serum *β* − *hCG* < 10 IU/L is certain to lead to failure to retrieve oocytes despite adequate follicular development and commensurate estradiol rise. Our case report suggests that the threshold below which final oocyte maturation would not be accomplished and therefore oocytes may not be retrieved is lower than once thought.

There are many factors that affect the pharmacokinetics of hCG. The half-life of hCG is affected by route of administration, BMI of the patient, and formulation of hCG [15, 16]. In this case, based on the contributing factors, it can be approximated that the half-life of hCG is 35 hours, and the drug would have reached its maximum concentration after 12 hours [16, 17]. Therefore, we can speculate that in our case when the serum *β*-hCG level was drawn 12 hours after administration, we were capturing the serum *β*-hCG level at its peak concentration of 16 IU/L. Furthermore, at the time of oocyte collection, which was 39 hours after hCG administration, the *β*-hCG level in vivo was likely ≤8 IU/L. Undoubtedly, the serum *β*-hCG concentrations that reach the follicles should be at a level capable of initiating meiosis and triggering the release of the cumulus-oocyte complex into the follicular fluid. However, there is currently no consensus on the optimal hCG dose requirements for initiating final oocyte maturation prior to oocyte collection in IVF; doses that range from 2,500 to 15,000 IU have been used [18–20] and often depended on the number of follicles that developed, the peak serum estradiol (E2) level, and the perceived risk of ovarian hyperstimulation syndrome (OHSS). In addition, Hoyos and colleagues [21] found that a lower dose of hCG (2,500 IU) was effective for oocyte maturation in IVF and that such a dose did not affect the clinical pregnancy and livebirth rates irrespective of patients' BMI. We could not find any report in the literature that advocates for the use of 2,000 IU hCG for ovulation trigger. Such low doses are only used for dual trigger with GnRh analogs to optimize clinical outcomes for high ovarian responders in GnRH-antagonist protocols [22].

Most cases of FEFS can be avoided by confirming that the patient self-administered her prescribed hCG dose by measuring the amount of *β*-hCG in her blood the morning after the hCG was administered, as is the practice in our unit and as advocated by others [9]. However, measuring the level of serum *β*-hCG on the day of retrieval and redosing when low and delaying the oocyte collection may result in spontaneous ovulation before the scheduled oocyte collection procedure if the patient had adequate serum *β*-hCG to complete final oocyte maturation as in the case presented. It is of note though that premature ovulation in cases of reported EFS even with GEFS have not been reported to date.

Our report suggests that the level of serum *β*-hCG below which oocytes will not be collected due to errors in hCG administration is lower than reported in the literature. This is true, given that the serum *β*-hCG 39 hours after ovulation triggering in our case is bound to be ≤8 IU/L; however, we did not draw blood to ascertain the serum *β*-hCG level at the time of oocyte retrieval. It is known that the prevalence of FEFS ranges from 0.07 to 0.32% [13]. The significance of this can be expressed by calculating the number needed to treat. Utilizing this method, 400 (NNT = 1/0.0007 − 0.0032) IVF cycles would need to be performed to prevent one case of FEFS. Therefore, it remains debatable whether this will be cost effective given the low incidence of the condition [9]. A positive urine pregnancy test may suffice, given that this reflects a serum *β*-hCG level of 20 IU/L [13].

As depicted by our case, there may be circumstances in which a rescue hCG dose may not be possible. In such cases, it may be beneficial to attempt oocyte retrieval in at least one ovary if serum *β*-hCG is equal to or greater than 16 IU/L the day before attempted or on the day of oocyte retrieval. If oocytes are retrieved, physicians should be reassured that completing the oocyte retrieval procedure will not compromise pregnancy rates. Even if oocytes are not retrieved after emptying a reasonable number of mature follicles, the retrieval can be abandoned, a rescue dose given, and the patient brought back 24-36 hours later for a second oocyte collection. This can be done even with normal bioavailability of *β*-hCG (≥40 IU/L) as with genuine empty follicle syndrome as advocated by Awonuga and colleagues [12], provided the oocyte collection is performed by an experienced operator.

It is also important to note that this experiment of nature became necessary not because it was planned but resulted from inadvertent administration of the wrong dose of hCG and our inability to obtain and administer a rescue hCG. This, however, has given us invaluable information as to the pharmacokinetics of hCG necessary for final oocyte maturation in ART. Notwithstanding the successful outcome of this case, the importance of patient education on correct hCG administration cannot be overemphasized, since this is a common cause of FEFS.

In conclusion, this is the first case reported in the literature in which a pregnancy resulted when the serum *β*-hCG level on the day before oocyte retrieval was 16 IU/L. This represents an opportunity for additional research to further understand the complexity of oocyte maturation and to pinpoint the lower threshold of serum *β*-hCG necessary for this process, as the level needed may be lower than previously thought.
